# Circulating retinol binding protein 4 levels in nonalcoholic fatty liver disease: a systematic review and meta-analysis

**DOI:** 10.1186/s12944-017-0566-7

**Published:** 2017-09-20

**Authors:** Zhongwei Zhou, Hongmei Chen, Huixiang Ju, Mingzhong Sun

**Affiliations:** Department of Clinical Laboratory, Affiliated Yancheng Hospital, School of Medicine, Southeast University, No. 75 Juchang Road, Tinghu, Yancheng, Jiangsu 224001 People’s Republic of China

**Keywords:** Retinol binding protein 4, Nonalcoholic fatty liver disease, Non-alcoholic steatohepatitis, Simple steatosis, Meta-analysis

## Abstract

**Background:**

Retinol binding protein 4 (RBP4) is implicated in obesity, insulin resistance and type 2 diabetes mellitus that are closely associated with nonalcoholic fatty liver disease (NAFLD). However, recent investigations regarding circulating RBP4 levels in NAFLD are conflicting. This meta-analysis is to determine whether NAFLD, non-alcoholic steatohepatitis (NASH) and simple steatosis (SS) patients have altered RBP4 levels.

**Methods:**

We performed a systematic search in PubMed, EMBASE and The Cochrane Library up until 18 March 2017, and 12 studies comprising a total of 4247 participants (2271 NAFLD patients and 1976 controls) were included in the meta-analysis.

**Results:**

There were no significant differences of circulating RBP4 levels in the following comparisons: (1) NAFLD patients vs controls (standardized mean differences [SMD]: 0.08; 95% CI: −0.21, 0.38); (2) NASH patients vs controls (SMD: −0.49; 95% CI: −1.09, 0.12); (3) SS patients vs controls (SMD: −0.72; 95% CI: −1.64, 0.20) and (4) NASH vs SS patients (SMD: −0.04; 95% CI: −0.32, 0.24). The results remained essentially unchanged in the comparisons between NAFLD patients and controls after excluding single individual study or bariatric studies (*n* = 2). No significant publication bias was detected. However, there was significant heterogeneity among studies and the subgroup and meta-regression analyses did not find the potential sources.

**Conclusions:**

Circulating RBP4 levels may not be associated with NAFLD. Further prospective cohort studies are required to confirm these findings.

**Electronic supplementary material:**

The online version of this article (10.1186/s12944-017-0566-7) contains supplementary material, which is available to authorized users.

## Background

Nonalcoholic fatty liver disease (NAFLD) has currently been one of the most common chronic liver disease, and its prevalence is about 25% worldwide [1]. The term ‘NAFLD’ comprises of a wide spectrum of hepatic histological changes ranging from simple steatosis (SS) to non-alcoholic steatohepatitis (NASH) and NASH-related fibrosis or cirrhosis [2]. Generally, NAFLD patients are more likely to be accompanied by obesity, insulin resistance, hyperglycemia, dyslipidemia, and hypertension, and therefore NAFLD is thought to be a hepatic manifestation of the metabolic syndrome [3].

Retinol binding protein 4 (RBP4) is a recently identified protein which belongs to the lipocalin family and is the specific carrier protein of vitamin A in the blood [4]. RBP4 is highly expressed in the liver, followed by adipose tissue [5]. Animal studies suggest that transgenic overexpression of human RBP4 or injection of recombinant RBP4 in wild-type mice causes insulin resistance; on the contrary, genetic deletion of RBP4 enhances insulin sensitivity [6]. In humans, a number of studies have shown that increased circulating RBP4 levels were correlated with obesity, insulin resistance, impaired glucose tolerance and type 2 diabetes mellitus (T2DM) [7–9]. Furthermore, decreased RBP4 levels have also been shown to be associated with improving insulin sensitivity after lifestyle intervention, weight loss or drug use [10–12].

Although the pathogenesis of NAFLD is not well known, insulin resistance has long been considered to play a key role in the development of NAFLD [13]. Therefore, NAFLD is supposed to be closely associated with increased RBP4 levels. However, studies of the association between circulating RBP4 levels and NAFLD yielded inconsistent findings. Some studies demonstrated that NAFLD patients had significantly increased RBP4 levels compared with healthy control individuals [14, 15], whereas other studies found no association between RBP4 levels and NAFLD [16, 17], Furthermore, some groups reported decreased circulating levels of RBP4 in patients with NAFLD [18, 19], Given the inconsistent reports, a systematic review and meta-analysis on this subject is warranted. In this study, we undertook what is, to our knowledge, the first systematic review and meta-analysis of studies on this subject aiming for getting a more persuasive conclusion.

## Methods

### Search strategy

Two independent investigators (Zhongwei Zhou and Hongmei Chen) performed a systematic search in PubMed, EMBASE and The Cochrane Library up until 18 March 2017, with no language restrictions. The search terms included: (“nonalcoholic fatty liver disease” OR NAFLD OR “nonalcoholic steatohepatitis” OR NASH OR steatohepat* OR steatosis OR “fatty liver*”) AND (“retinol-binding protein-4” OR RBP4). In addition, we examined the reference lists in relevant original research and review articles to search additional potentially eligible studies.

### Inclusion and exclusion criteria

Studies reporting circulating RBP4 levels in patients with NAFLD were eligible for review. Additional inclusion criteria were (1) studies in adults subjects (age ≥ 18 years); (2) studies comparing RBP4 levels in patients with NAFLD (or SS or NASH) with healthy control subjects; or (3) studies comparing RBP4 levels in different stages of the diseases (ie, comparison between SS and NASH). The search was not limited by language or publication time. In studies that had more than one control group, we included the control group whose characteristics were more similar to the case group, and in this study, we adopted body mass index (BMI) matched strategy.

The papers were excluded from the systematic review and meta-analysis if (1) studies that examined other types of liver disease (ie, alcoholic fatty liver disease, viral or autoimmune hepatitis); (2) studies that were interventional with similar groups at baseline; (3) samples that overlapped with another study; or (4) studies that were reviews, case reports, letters to the editor, comment, studies on animals or cell lines, conference abstracts, or unpublished studies.

### Data extraction

Data was extracted independently by two investigators (Zhongwei Zhou and Hongmei Chen) and confirmed by a third reviewer (Mingzhong Sun). Disagreement was resolved by discussion among all researchers. If necessary data were not offered, the corresponding authors were contacted. When the corresponding authors did not respond, transformations were made by standard formulas. If study populations overlapped, the study reporting the largest sample was included. We abstracted the following information from each selected publication: (1) the study’s general characteristics such as first author’s name, year of publication, country where the study was carried out, study design, the diagnostic methods of NAFLD; (2) subjects characteristics such as age, gender, BMI and the number of subjects with T2DM; (3) the biochemical measurements of subjects including aspartate aminotransferase (AST), alanine aminotransferase (ALT), γ-glutamyltranspeptidase (GGT) and RBP4 levels; and (4) the evaluation of insulin resistance by homoeostasis model assessment of insulin resistance (HOMA-IR).

### Quality assessment

The included studies in the systematic review and meta-analysis were independently assessed by two investigators (Zhongwei Zhou and Hongmei Chen). The assessment was based on the modified Newcastle-Ottawa Quality Assessment Scale (NOS) suggested by van et al. [20]. The full score was 9 stars, and a study that met 7 or more stars was defined as a high-quality study, less than 3 stars low-quality study, and other studies were defined as moderate quality.

### Statistical analysis

All statistical analyses were performed using Stata14.0 (StataCorp LP, College Station, TX, USA). The effect sizes were generated by sample sizes, mean RBP4 levels, and the standard deviation (SD), and presented as standardized mean differences (SMD) and 95% confidence interval (CI) in RBP4 levels in comparisons between groups. A random-effect model was chosen for this meta-analysis, because this model is a more conservative approach which yields a wider CI than fixed effect model if there is a significant heterogeneity between included studies [21].

The heterogeneity between the results of different studies was evaluated using the *I* ^2^ statistic, and an *I* ^2^ values of 25%, 50% and 75% would indicate low, moderate and high heterogeneity, respectively [22]. The potential moderating effects of continuous variables on between-study heterogeneity were evaluated by meta-regression analyses. We assumed sample size, sex distribution (the number of males), mean age, BMI, HOMA-IR and ALT levels of NAFLD patients as potential moderators for the outcome of the meta-analysis.

Sensitivity analysis was applied to evaluate the influence of each study on the pooled measures by omitting one in turn and recalculating the pooled SMD for the remainders. We also performed a test by excluding studies reporting on morbidly obese populations subjected to bariatric surgery, which was used as another sensitivity analysis. Publication bias was assessed by Funnel’s plot and Egger’s test.

## Results

### Literature search

**Fig. 1:**
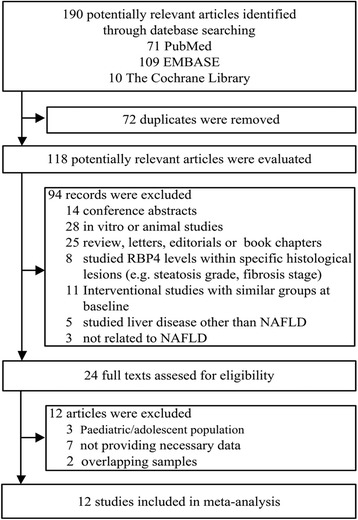
Flow chart of the study selection process

We initially retrieved 190 articles from three databases including PubMed, EMBASE and The Cochrane Library. After reading the titles and abstracts, 24 appropriate articles were identified for full-text analysis. The 12 articles were further excluded for studies that limited in Paediatric/adolescent population, lack of necessary data on RBP4 levels and patient samples that overlapped with another study. Thus, 12 studies met the criteria for inclusion in the present meta-analysis [14–19, 23–28], and a flowchart of the included and excluded studies was shown in Fig. 1.

### Characteristics of the included studies

The 12 included studies were published from 2008 to 2017 covering 2271 NAFLD patients and 1976 controls. The main characteristics of these studies are presented in Table 1. Six studies were carried out in Asia, three in Europe, two in USA and one in Australia. All studies included in this meta-analysis were cross-sectional. The method of RBP4 measurement was ELISA in all of the studies except for one which was Radioimmunoassay [26]. Liver biopsy was performed for determining NAFLD in six studies, ultrasound techniques in five studies, a combination of both in one study.

**Table 1:** Main characteristics of the studies included in this meta-analysis

References	Study location	Study design	Method of RBP4 measurement	Diagnostic methods of NAFLD	Quality Score	Additional information
Seo et al. [14]	Korea	Cross-sectional	ELISA	Ultrasound	3	
Chen et al. [15]	China	Cross-sectional	ELISA	Ultrasound	3	
Milner et al. [16]	Australia	Cross-sectional	ELISA	Liver biopsy	7	
Suh et al. [17]	Korea	Cross-sectional	ELISA	Ultrasound	4	
Schina et al. [18]	Greece	Cross-sectional	ELISA	Liver biopsy	4	
Polyzos et al. [19]	Greece	Cross-sectional	ELISA	Liver biopsy	5	A second control group of 24 lean subjects was not included in the meta-analysis.
Auguet et al. [23]	Spain	Cross-sectional	ELISA	Liver biopsy	6	Patients and controls were morbidly obese women who underwent bariatric surgery.
Koh et al. [24]	Korea	Cross-sectional	ELISA	Ultrasound	5	NAFLD cases and controls were all type 2 diabetic patients.
Wu et al. [25]	China	Cross-sectional	Radioimmunoa- ssay	Ultrasound	3	NAFLD cases and controls were all type 2 diabetic patients.
Cengiz et al. [26]	Turkey	Cross-sectional	ELISA	Ultrasound and liver biopsy	3	34 of 76 NAFLD underwent a liver biopsy.
Kashyap et al. [27]	USA	Cross-sectional	ELISA	Liver biopsy	6	Patients and controls were subjected to bariatric surgery
Alkhouri et al. [28]	USA	Cross-sectional	ELISA	Liver biopsy	4	

*RBP4* retinol binding protein 4, *NAFLD* nonalcoholic fatty liver disease

The main demographic and biochemical characteristics of studies included in this meta-analysis are presented in Additional file 1: Table S1. We reported circulating RBP4 levels, age, the number of (males) subjects and T2DM patients, BMI, HOMA-IR and liver enzyme (AST, ALT and GGT) levels. Four studies included all groups (control, SS, NASH and NAFLD) [16, 18, 19, 27]; seven studies compared RBP4 levels between NAFLD patients and controls, without providing separate data for SS and NASH [14, 15, 17, 23–26] and one study compared RBP4 levels between SS and NASH patients, without recruiting a control group [28], Then, comparative data was provided as follows: 11 studies, NAFLD patients (*n* = 2222) vs controls (*n* = 1976); four studies, NASH patients (*n* = 128) vs controls (*n* = 230); four studies, SS patients (*n* = 92) vs controls (n = 230) and 5 studies, NASH (*n* = 161) vs SS patients (*n* = 108).

### Quality of included studies

We assessed the quality of included studies based on the modified NOS. All the studies were assessed as moderate quality except for one which was assessed as high quality. Quality score of each study was exhibited in Table 1.

### Meta-analysis

**Fig. 2:**
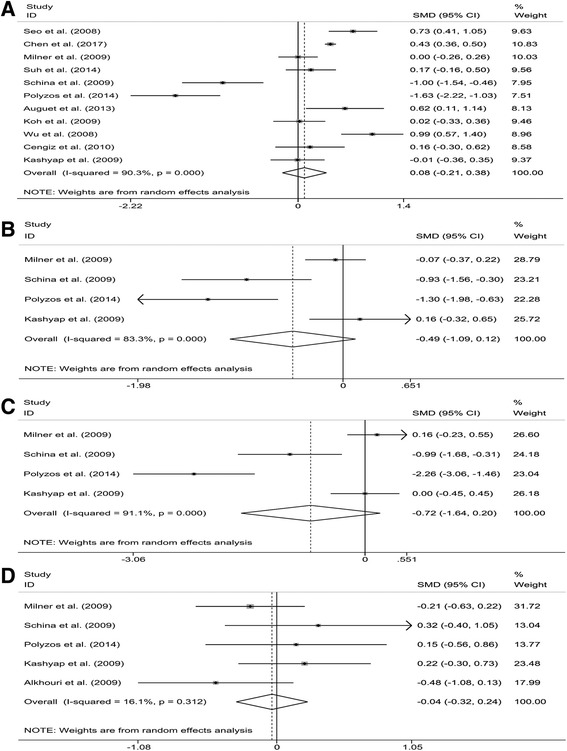
Meta-analysis of circulating retinol binding protein 4 (RBP4) levels in nonalcoholic fatty liver disease (NAFLD) patients compared with controls (**a**), non-alcoholic steatohepatitis (NASH) patients compared with controls (**b**), simple steatosis (SS) patients compared with controls (**c**) and NASH patients compared with SS patients (**d**). SMD, standardized mean differences; CI, confidence interval

**Fig. 3:**
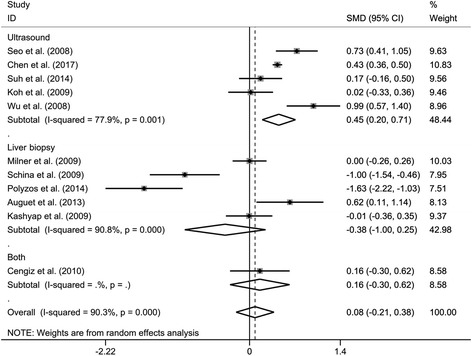
Subgroup analysis of circulating retinol binding protein 4 (RBP4) levels in nonalcoholic fatty liver disease (NAFLD) patients compared with controls when stratified by the diagnostic methods of NAFLD. SMD, standardized mean differences; CI, confidence interval

We performed a random-effects meta-analysis on the extracted 12 studies. The results showed that there were no significant differences of circulating RBP4 levels in the following comparisons: (1) NAFLD patients vs controls (SMD: 0.08; 95% CI: −0.21, 0.38; *P* = 0.568) (Fig. 2a); (2) NASH patients vs controls (SMD: −0.49; 95% CI: −1.09, 0.12; *P* = 0.116) (Fig. 2b); (3) SS patients vs controls (SMD: −0.72; 95% CI: −1.64, 0.20; *P* = 0.125) (Fig. 2c) and (4) NASH vs SS patients (SMD: −0.04; 95% CI: −0.32, 0.24; *P* = 0.791) (Fig. 2d). However, significant heterogeneity among studies was found in comparisons of NAFLD vs controls, NASH vs controls and SS vs controls, with *I* ^2^ values 90.3%, 83.3% and 91.1%, respectively; all *P* < 0.001) (Fig. 2a–c).

### Investigation of heterogeneity

Owing to the small number of studies in comparisons of NASH vs controls (*n* = 4) and SS vs controls (n = 4), an exploration of heterogeneity was only performed in studies that compared NAFLD patients and healthy controls (*n* = 11).

To investigate whether the different methods of determining NAFLD could explain the high levels of heterogeneity, we performed subgroup analysis according to the diagnostic methods of NAFLD. As shown in Fig. 3, the NAFLD patients who were diagnosed by ultrasound techniques had significantly increased RBP4 levels compared with healthy controls (SMD: 0.45; 95% CI: 0.20, 0.71; *P* = 0.001). In contrast, the patients diagnosed by liver biopsy did not show a difference in RBP4 levels compared with healthy controls (SMD: −0.38; 95% CI: −1.00, 0.25; *P* = 0.237), and nor did the patients diagnosed by both methods (SMD: 0.16; 95% CI: −0.30, 0.62; *P* = 0.498). However, high levels of heterogeneity were still found in studies of ultrasound techniques (*I* ^2^ = 77.9%; *P* = 0.001) and liver biopsy (*I* ^2^ = 90.8%; *P* < 0.001).

We next performed univariate, random-effects meta-regression analyses to test whether the continuous variables, including sample size, the number of males, mean age, BMI, HOMA-IR and ALT levels of NAFLD patients, could explain the high levels of heterogeneity among studies. We found all the tested variables did not show moderating effects on the outcome of the meta-analysis (Additional file 2: Table S2).

### Sensitivity and publication bias analyses

Sensitivity and publication bias analyses were performed also only in studies that compared NAFLD patients and healthy controls.

Sensitivity analysis indicated that no individual study significantly influenced the difference on RBP4 levels between patients with NAFLD and healthy controls (Additional file 3: Figure S1). In addition, after excluding bariatric studies (*n* = 2), there were only minimal changes in the comparisons between NAFLD patients and controls (SMD: 0.04; 95% CI: **-**0.30, 0.38; *P* = 0.831) (Additional file 4: Figure S2).

Visual inspection of funnel plots (Additional file 5: Figure S3) showed no significant publication bias in this meta-analysis, which was further confirmed by Begg’s and Egger’s test (*P* = 0.120).

## Discussion

In this meta-analysis, we found NAFLD, NASH or SS patients did not show significant differences of circulating RBP4 levels when compared with the controls, and NASH patients also had similar RBP4 levels with SS patients. The sensitivity analyses did not essentially influence these findings. In addition, we did not find significant publication bias in this meta-analysis.

Although circulating RBP4 levels were not associated with NAFLD overall, the results of subgroup analysis suggested that RBP4 levels were significantly higher in NAFLD patients who were diagnosed by ultrasound techniques compared with the controls. In this meta-analysis, ultrasound techniques were performed for determining NAFLD in five studies, in which three studies [14, 15, 25] reported that circulating RBP4 levels were significantly increased in NAFLD patients compared with controls. But we found all the three studies were assessed with lower quality score (NOS Quality Score = 3), and one study [15] in which the age and BMI of patients were not matched to the controls, and one study [25] in which NAFLD cases and controls were all T2DM patients. Moreover, though ultrasound examination has been proven to be an reliable imaging technique for the detection of NAFLD, conventional ultrasound B-mode imaging which was used in all the five studies is mainly qualitative in nature resulting in lack of specificity and sensitivity, and the new quantitative ultrasound technique has not been in widespread use in the detection of NAFLD [29, 30]. Therefore, more high-quality research and better imaging technologies such as quantitative ultrasound are required to assess whether circulating RBP4 levels were increased in NAFLD patients diagnosed by noninvasive technique.

In the meta-analysis, we analyzed the potential factors contributing to heterogeneity only in the comparison of NAFLD vs controls because of the limited amount of studies in other comparisons. When subgroup analysis was performed, although the heterogeneity was slightly reduced in the ultrasound group, substantial between-study heterogeneity was still found in the group of liver biopsy. Meta-regression analyses indicated that sample size, the number of males, mean age, BMI, HOMA-IR and ALT levels of NAFLD patients were all not confounding factors which accounted for the heterogeneity among eligible studies. Because of limited information available from the included studies, we are not sure whether some other factors could contribute to the between-study heterogeneity. Recently, it has been proved that the genetic variant I148M (rs738409) in patatin-like phospholipase domain-containing protein 3 (PNPLA3) was a common mutation and associated with chronic liver disease [31, 32], and carriers of the PNPLA3 148 M allele in obese individuals with or without NAFLD had lower circulating RBP4 concentrations [33]. In this meta-analyses, however, all of the studies included did not exclude the influence of PNPLA3 I148M Variant on circulating RBP4 levels. In addition, a number of studies have indicated that circulating RBP4 concentrations were associated with renal dysfunction [34–36], and NAFLD has been found to be associated with decreased estimated glomerular filtration rate (eGFR) and/or microalbuminuria [37, 38]. Although some of the studies in this meta-analysis referred to the exclusion criteria that did not include subjects with renal dysfunction, we are not sure if it was strictly controlled, and some of the studies did not refer to the exclusion. Some potential moderators such as exercise, diet adjustment, and drug use that had influence upon the expression of circulating RBP4 levels [12, 39, 40] were also limited in the eligible studies included in the meta-analysis, which prevented us from analyzing whether these factors had moderating effects on the results. Some technical factor which may account for the between-study heterogeneity should also be considered. For example, some studies collected blood samples in a fasting state, and others provided no information; the storage temperature of blood samples differed in different studies, ranging from −20 °C to −80 °C, but some studies did not provide the information.

The limitations of this study should also be of concern. First, considerable heterogeneity among studies limits the reliability of the results. Although we performed subgroup and meta-regression analyses to investigate some potential sources, the high levels of heterogeneity cannot be reasonably explained. Therefore, the results of this meta-analysis should be cautiously interpreted. Second, liver biopsy was considered the gold standard for diagnosing NAFLD [41], which was used only in half of studies in this meta-analysis. However, we stratified our analyses by the diagnostic method and found that the results of the overall meta-analysis were consistent with the results of stratification in liver biopsy. Third, our assessment of study quality was based on the modified NOS owing to a lack of appropriate quality-assessment tool for cross-sectional studies, which may lead to arbitrary results [42].

## Conclusions

In conclusion, circulating RBP4 levels may not be associated with NAFLD, which suggests it might not be potential non-invasive biomarkers for identifying NAFLD. However, the results should be cautiously interpreted because of the substantial unexplained between-study heterogeneity, and further prospective cohort studies are required to confirm these findings.

## Additional files

Additional file 1: **Table S1.** Main demographic and biochemical characteristics of the studies included in this meta-analysis. (DOCX 29 kb)

Additional file 2: **Table S2.** Meta-regression analysis to assess the influence of continuous variables on the effect sizes in studies that compared nonalcoholic fatty liver disease (NAFLD) patients and healthy controls. (DOCX 17 kb)

Additional file 3: **Figure S1.** Sensitivity analysis of included studies for the influence of circulating retinol binding protein 4 (RBP4) levels between nonalcoholic fatty liver disease (NAFLD) patients and controls. CI, confidence interval. (TIFF 271 kb)

Additional file 4: **Figure S2.** Meta-analysis of circulating retinol binding protein 4 (RBP4) levels in nonalcoholic fatty liver disease (NAFLD) patients compared with controls after excluding studies on morbidly obese individuals subjected to bariatric surgery. SMD, standardized mean differences; CI, confidence interval. (TIFF 263 kb)

Additional file 5: **Figure S3.** Begg’s funnel plot of included studies for potential publication bias between nonalcoholic fatty liver disease (NAFLD) patients and controls. SMD, standardized mean differences. (TIFF 111 kb)
