# Supplementary material for: Circulating retinol binding protein 4 levels in nonalcoholic fatty liver disease: a systematic review and meta-analysis
Source: Lipids Health Dis. 2017 Sep 20;16:180. doi: 10.1186/s12944-017-0566-7 (PMC5607593; doi:10.1186/s12944-017-0566-7)
Supplement: Supplementary file 1 — Table S1. Main demographic and biochemical characteristics of the studies included in this meta-analysis. (DOCX 29 kb) [file 12944_2017_566_MOESM1_ESM.docx]

Table 1_SuppInfo Main demographic and biochemical characteristics of the studies included in this meta-analysis

| References | Group | N (Males) | Age  (years) | T2DM  (N) | BMI  (kg/m^2^) | AST  (IU/L) | ALT  (IU/L) | GGT  (IU/L) | HOMA-IR | RBP4  (ug/mL) |
| --- | --- | --- | --- | --- | --- | --- | --- | --- | --- | --- |
| Seo et al. [14] | Control | 86 (50) | 49.7±10.1 | na | 23.7±2.4 | 19.4±1.2 | 15.4±1.5 | 21.6±1.7 | 1.6±1.4 | 51.7±14.6 |
|  | SS |  |  |  |  |  |  |  |  |  |
|  | NASH |  |  |  |  |  |  |  |  |  |
|  | NAFLD | 73 (45) | 49.6±12.5 | na | 27±2.3 | 25.8±1.4 | 30.9±1.7 | 41±1.9 | 2.5±1.5 | 62.8±16.0 |
| Chen et al. [15] | Control | 1458 (436) | 60.5±6.1 | 93 | 22.1±2.6 | 21.1±4.6 | 15.7±5.4 | na | 1.43±0.71 | 35.0±6.7 |
|  | SS |  |  |  |  |  |  |  |  |  |
|  | NASH |  |  |  |  |  |  |  |  |  |
|  | NAFLD | 1480 (483) | 60.8±5.6 | 140 | 25.0±3.0 | 19.7±5.0 | 19.0±7.6 | na | 2.47±1.28 | 37.9±6.8 |
| Milner et al. [16] | Control | 129 (71) | 47.4±10.7 | 0 | 26.9±4.8 | na | 24.6±9.9 | na | 1.9±1.1 | 16.3±3.8 |
|  | SS | 31 (22) | 44.5±12.6 | 6 | 29.6±4.9 | na | 66.6±34 | na | 3.6±1.8 | 16.9±3.6 |
|  | NASH | 69 (38) | 49.8±11.5 | 22 | 31.0±5.0 | na | 82.1±53.8 | na | 6.3±6.5 | 16.0±4.6 |
|  | NAFLD | 100 (60) | 48.2±12.0 | 28 | 30.6±5.0 | na | 77.3±48.9 | na | 5.4±5.6 | 16.3±3.8 |
| Suh et al. [17] | Control | 67 (38) | 49.5±10.4 | na | 24.4±2.4 | 21.3±6.5 | 20.0±8.5 | 22.4±11.8 | 2.5±1.1 | 54.7±27.7 |
|  | SS |  |  |  |  |  |  |  |  |  |
|  | NASH |  |  |  |  |  |  |  |  |  |
|  | NAFLD | 73 (34) | 50.5±9.7 | na | 25.5±3.0 | 25.7±13.7 | 31.6±22.7 | 36.0±31.4 | 3.0±1.3 | 60.2±35.8 |
| Schina et al. [18] | Control | 30 (13) | 49.0±16.2 | na | 24.6±3.3 | na | 28±9.6 | na | 2.4±0.5 | 34.7±12.3 |
|  | SS | 13 (5) | 53.0±12.7 | na | 27.5±4.4 | na | 44±39.3 | na | 3.2±2.2 | 22.9±10.0 |
|  | NASH | 17 (13) | 50.1±16.6 | na | 30.2±3.8 | na | 58±49.6 | na | 3.9±2.9 | 25.2±3.0 |
|  | NAFLD | 30 (18) | 51.0±14.9 | na | 28.6±4.7 | na | 55±36.3 | na | 3.5±2.3 | 25.2±5.0 |
| Polyzos et al. [19] | Control | 28 (8) | 52.6±1.6 | na | 30.9±0.6 | 19±1 | 20±2 | 21±3 | 2.25±0.25 | 0.87±0.08 |
|  | SS | 15 (5) | 53.9±2.6 | na | 31.9±1.3 | 27±2 | 42±6 | 46±12 | 5.34±2.52 | 0.65±0.12 |
|  | NASH | 16 (3) | 53.9±2.9 | na | 34.1±1.4 | 49±9 | 71±15 | 64±12 | 5.78±1.14 | 0.73±0.14 |
|  | NAFLD | 31 (8) | 53.9±2.7 | na | 33.0±1.7 | 38±13 | 57±19 | 55±15 | 5.57±1.91 | 0.69±0.13 |
| Auguet et al. [23] | Control | 19 (0) | 44.1±10.7 | na | 49.5±7.0 | 31.6±16.1 | 30.5±14.3 | 19.8±11.6 | 2.7±1.6 | 26.0±7.0 |
|  | SS |  |  |  |  |  |  |  |  |  |
|  | NASH |  |  |  |  |  |  |  |  |  |
|  | NAFLD | 69 (0) | 46.8±10.3 | na | 48.2±6.6 | 48.9±32.3 | 50.7±30.8 | 38.0±37.4 | 2.9±2.6 | 39.0±23.0 |
| Koh et al. [24] | Control | 42 (27 ) | 53.4±10.5 | 42 | 24.0±2.3 | 20.5±6.5 | 23.5±10.2 | 27.8±18.5 | 3.1±3.5 | 72.7±28.7 |
|  | SS |  |  |  |  |  |  |  |  |  |
|  | NASH |  |  |  |  |  |  |  |  |  |
|  | NAFLD | 139 (73) | 54.6±10.6 | 139 | 26.2±3.5 | 29.4±16.0 | 37.6±24.1 | 47.1±39.2 | 3.85±3.1 | 73.2±29.8 |
| Wu et al. [25] | Control | 50 (30) | 52.8±7.1 | 50 | 24.6±3.4 | na | 23.7±14.0 | 35.6±24.3 | 5.8±3.0 | 32.0±8.9 |
|  | SS |  |  |  |  |  |  |  |  |  |
|  | NASH |  |  |  |  |  |  |  |  |  |
|  | NAFLD | 52 (31) | 53.6±9.8 | 52 | 25.8±2.7 | na | 36.1±20.3 | 61.2±48.5 | 7.1±3.3 | 41.3±9.8 |
| Cengiz et al. [26] | Control | 24 (na) | 38±10 | na | 25.6±1.1 | na | 28.8±7.6 | 28.5±7.5 | 1.60±0.47 | 18.0±0.7 |
|  | SS |  |  |  |  |  |  |  |  |  |
|  | NASH |  |  |  |  |  |  |  |  |  |
|  | NAFLD | 76 (na) | 39±9 | na | 30.1±4.5 | na | 81.4±58.9 | 63.3±43.2 | 3.63±3.24 | 18.1±0.6 |
| Kashyap et al. [27] | Control | 43 (6) | 48.6±10.7 | 15 | 48.7±6.8 | 21.3±6.6 | 19.4±9.5 | na | na | 41.6±21.2 |
|  | SS | 33 (10) | 46.3±9.9 | 12 | 48.7±7.8 | 21.5±6.7 | 24.0±11.0 | na | na | 41.6±13.6 |
|  | NASH | 26 (5) | 47.1±12.1 | 11 | 50.4±11.5 | 44.6±41.3 | 48.8±42.6 | na | na | 44.8±15.8 |
|  | NAFLD | 99 (24) | 48.4±10.6 | 40 | 49.0±9.0 | 28.5±23.8 | 31.5±25.8 | na | na | 41.5±13.2 |
| Alkhouri et al. [28] | Control |  |  |  |  |  |  |  |  |  |
|  | SS | 16 (9) | 45.1±11 | na | 30.4±5.3 | 50.0±40.4 | 41.5±23.7 | na | 1.8±1.4 | 26.9±13.4 |
|  | NASH | 33 (15) | 51.1±10 | na | 33.2±4.6 | 84.5±48.5 | 62.5±28.2 | na | 6.9±7.9 | 21.4±10.3 |
|  | NAFLD |  |  |  |  |  |  |  |  |  |

Data are presented as mean±standard deviation or absolute frequencies. When the data were not presented as mean±standard deviation in the original, they were properly transformed to. T2DM, type 2 diabetes mellitus; BMI, body mass index; AST, aspartate aminotransferase; ALT, alanine aminotransferase; GGT, γ-glutamyltranspeptidase; HOMA-IR, homoeostasis model assessment of insulin resistance; RBP4, retinol binding protein 4; na, not available; SS, simple steatosis; NASH, non-alcoholic steatohepatitis; NAFLD, nonalcoholic fatty liver disease.

**References**

14. Seo JA, Kim NH, Park SY, Kim HY, Ryu OH, Lee KW, Lee J, Kim DL, Choi KM, Baik SH, Choi DS, Kim SG. Serum retinol-binding protein 4 levels are elevated in non-alcoholic fatty liver disease. Clin Endocrinol (Oxf). 2008;68**:**555-60.

15. Chen X, Shen T, Li Q, Chen X, Li Y, Li D, Chen G, Ling W, Chen YM. Retinol Binding Protein-4 Levels and Non-alcoholic Fatty Liver Disease: A community-based cross-sectional study. Sci Rep. 2017;7**:**45100.

16. Milner KL, van der Poorten D, Xu A, Bugianesi E, Kench JG, Lam KS, Chisholm DJ, George J. Adipocyte fatty acid binding protein levels relate to inflammation and fibrosis in nonalcoholic fatty liver disease. Hepatology. 2009;49**:**1926-34.

17. Suh JB, Kim SM, Cho GJ, Choi KM. Serum AFBP levels are elevated in patients with nonalcoholic fatty liver disease. Scand J Gastroenterol. 2014;49**:**979-85.

18. Schina M, Koskinas J, Tiniakos D, Hadziyannis E, Savvas S, Karamanos B, Manesis E, Archimandritis A. Circulating and liver tissue levels of retinol-binding protein-4 in non-alcoholic fatty liver disease. Hepatol Res. 2009;39**:**972-8.

19. Polyzos SA, Kountouras J, Anastasilakis AD, Geladari EV, Mantzoros CS. Irisin in patients with nonalcoholic fatty liver disease. Metabolism. 2014;63**:**207-17.

20. van Dijk GM, Maneva M, Colpani V, Dhana K, Muka T, Jaspers L, Kavousi M, Franco OH. The association between vasomotor symptoms and metabolic health in peri- and postmenopausal women: a systematic review. Maturitas. 2015;80**:**140-7.

21. Masi A, Quintana DS, Glozier N, Lloyd AR, Hickie IB, Guastella AJ. Cytokine aberrations in autism spectrum disorder: a systematic review and meta-analysis. Mol Psychiatry. 2015;20**:**440-6.

22. Ioannidis JP, Patsopoulos NA, Evangelou E. Uncertainty in heterogeneity estimates in meta-analyses. BMJ. 2007;335**:**914-6.

23. Auguet T, Terra X, Porras JA, Orellana-Gavalda JM, Martinez S, Aguilar C, Lucas A, Pellitero S, Hernandez M, Del Castillo D, Richart C. Plasma visfatin levels and gene expression in morbidly obese women with associated fatty liver disease. Clin Biochem. 2013;46**:**202-8.

24. Koh JH, Shin YG, Nam SM, Lee MY, Chung CH, Shin JY. Serum adipocyte fatty acid-binding protein levels are associated with nonalcoholic fatty liver disease in type 2 diabetic patients. Diabetes Care. 2009;32**:**147-52.

25. Wu H, Jia W, Bao Y, Lu J, Zhu J, Wang R, Chen Y, Xiang K. Serum retinol binding protein 4 and nonalcoholic fatty liver disease in patients with type 2 diabetes mellitus. Diabetes Res Clin Pract. 2008;79**:**185-90.

26. Cengiz C, Ardicoglu Y, Bulut S, Boyacioglu S. Serum retinol-binding protein 4 in patients with nonalcoholic fatty liver disease: does it have a significant impact on pathogenesis? Eur J Gastroenterol Hepatol. 2010;22**:**813-9.

27. Kashyap SR, Diab DL, Baker AR, Yerian L, Bajaj H, Gray-McGuire C, Schauer PR, Gupta M, Feldstein AE, Hazen SL, Stein CM. Triglyceride levels and not adipokine concentrations are closely related to severity of nonalcoholic fatty liver disease in an obesity surgery cohort. Obesity (Silver Spring). 2009;17**:**1696-701.

28. Alkhouri N, Lopez R, Berk M, Feldstein AE. Serum retinol-binding protein 4 levels in patients with nonalcoholic fatty liver disease. J Clin Gastroenterol. 2009;43**:**985-9.
