# Supplementary material for: Circulating retinol binding protein 4 levels in nonalcoholic fatty liver disease: a systematic review and meta-analysis
Source: Lipids Health Dis. 2017 Sep 20;16:180. doi: 10.1186/s12944-017-0566-7 (PMC5607593; doi:10.1186/s12944-017-0566-7)
Supplement: Supplementary file 2 — Table S2. Meta-regression analysis to assess the influence of continuous variables on the effect sizes in studies that compared nonalcoholic fatty liver disease (NAFLD) patients and healthy controls. (DOCX 17 kb) [file 12944_2017_566_MOESM2_ESM.docx]

Table 2_SuppInfo Meta-regression analysis to assess the influence of continuous variables on the effect sizes in studies that compared nonalcoholic fatty liver disease (NAFLD) patients and healthy controls

| Variables (NAFLD patients) | Adjusted *R*^2^ (%) | *β* | 95% CI | *P* |
| --- | --- | --- | --- | --- |
| Sampe size | −9.29 | 1.000 | 0.999-1.001 | 0.563 |
| The number of males | −9.72 | 1.001 | 0.996-1.005 | 0.554 |
| Mean age | −13.59 | 0.992 | 0.897-1.097 | 0.861 |
| Mean BMI | −13.00 | 0.996 | 0.934-1.062 | 0.888 |
| Mean HOMA-IR levels | −12.25 | 0.927 | 0.618-1.393 | 0.681 |
| Mean ALT levels | 3.16 | 0.987 | 0.962-1.012 | 0.287 |

CI, confidence interval; BMI, body mass index; HOMA-IR, homoeostasis model assessment of insulin resistance; ALT, alanine aminotransferase.
