# Supplementary figures and images for: Circulating retinol binding protein 4 levels in nonalcoholic fatty liver disease: a systematic review and meta-analysis
Source: Lipids Health Dis. 2017 Sep 20;16:180. doi: 10.1186/s12944-017-0566-7 (PMC5607593; doi:10.1186/s12944-017-0566-7)

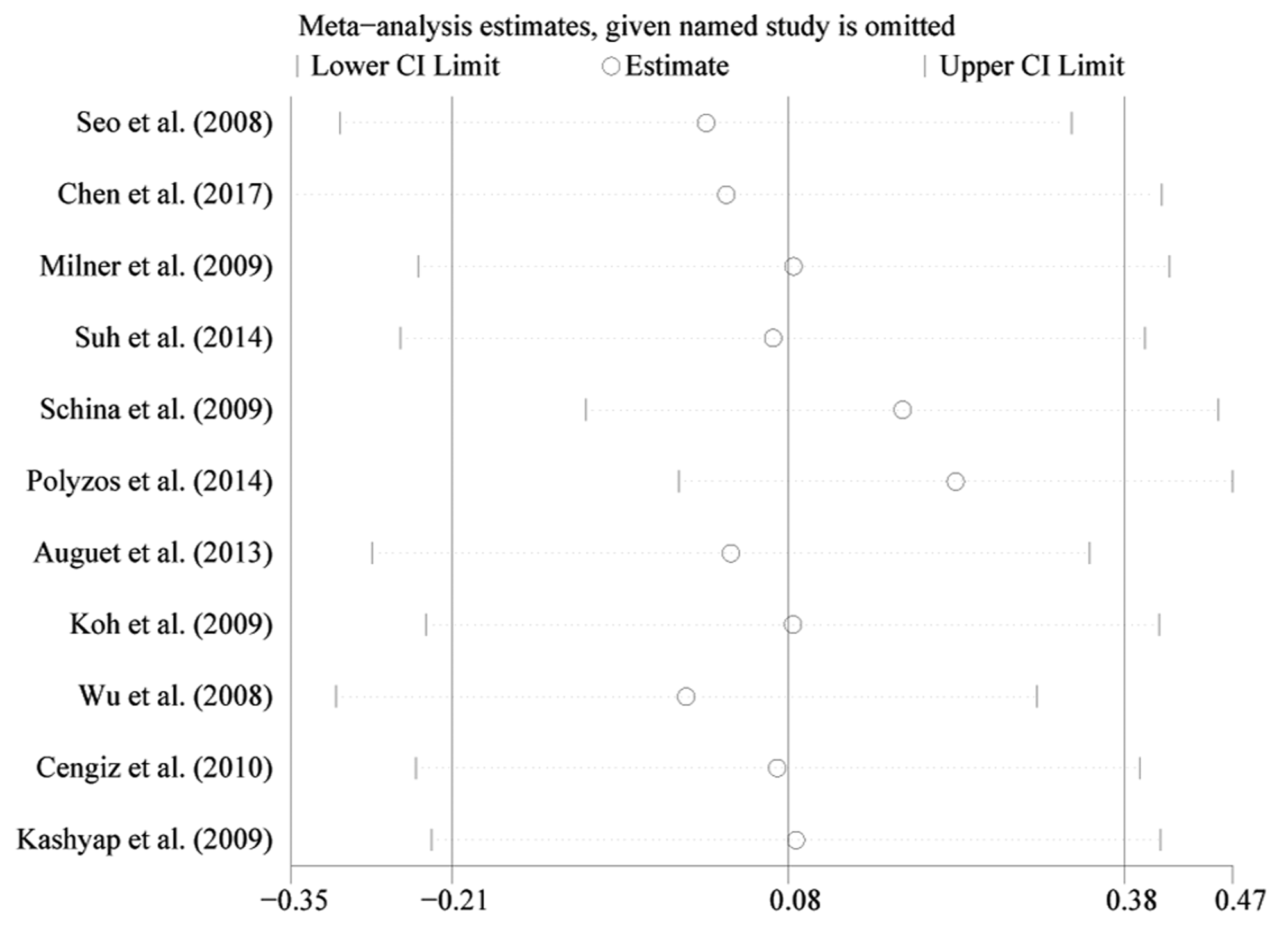

Supplement: Supplementary file 3 — Figure S1. Sensitivity analysis of included studies for the influence of circulating retinol binding protein 4 (RBP4) levels between nonalcoholic fatty liver disease (NAFLD) patients and controls. CI, confidence interval. (TIFF 271 kb) [file 12944_2017_566_MOESM3_ESM.tif]

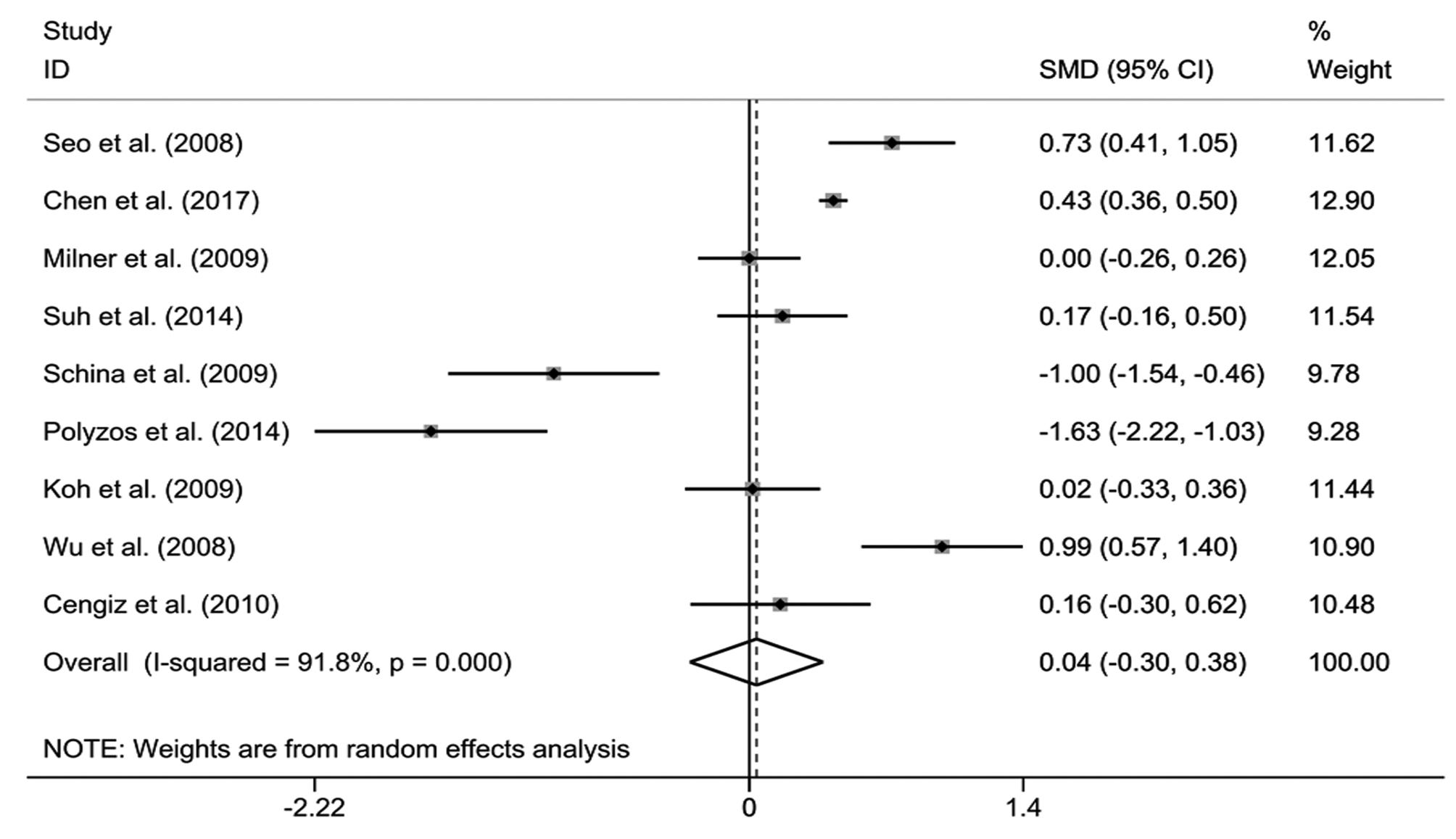

Supplement: Supplementary file 4 — Figure S2. Meta-analysis of circulating retinol binding protein 4 (RBP4) levels in nonalcoholic fatty liver disease (NAFLD) patients compared with controls after excluding studies on morbidly obese individuals subjected to bariatric surgery. SMD, standardized mean differences; CI, confidence interval. (TIFF 263 kb) [file 12944_2017_566_MOESM4_ESM.tif]

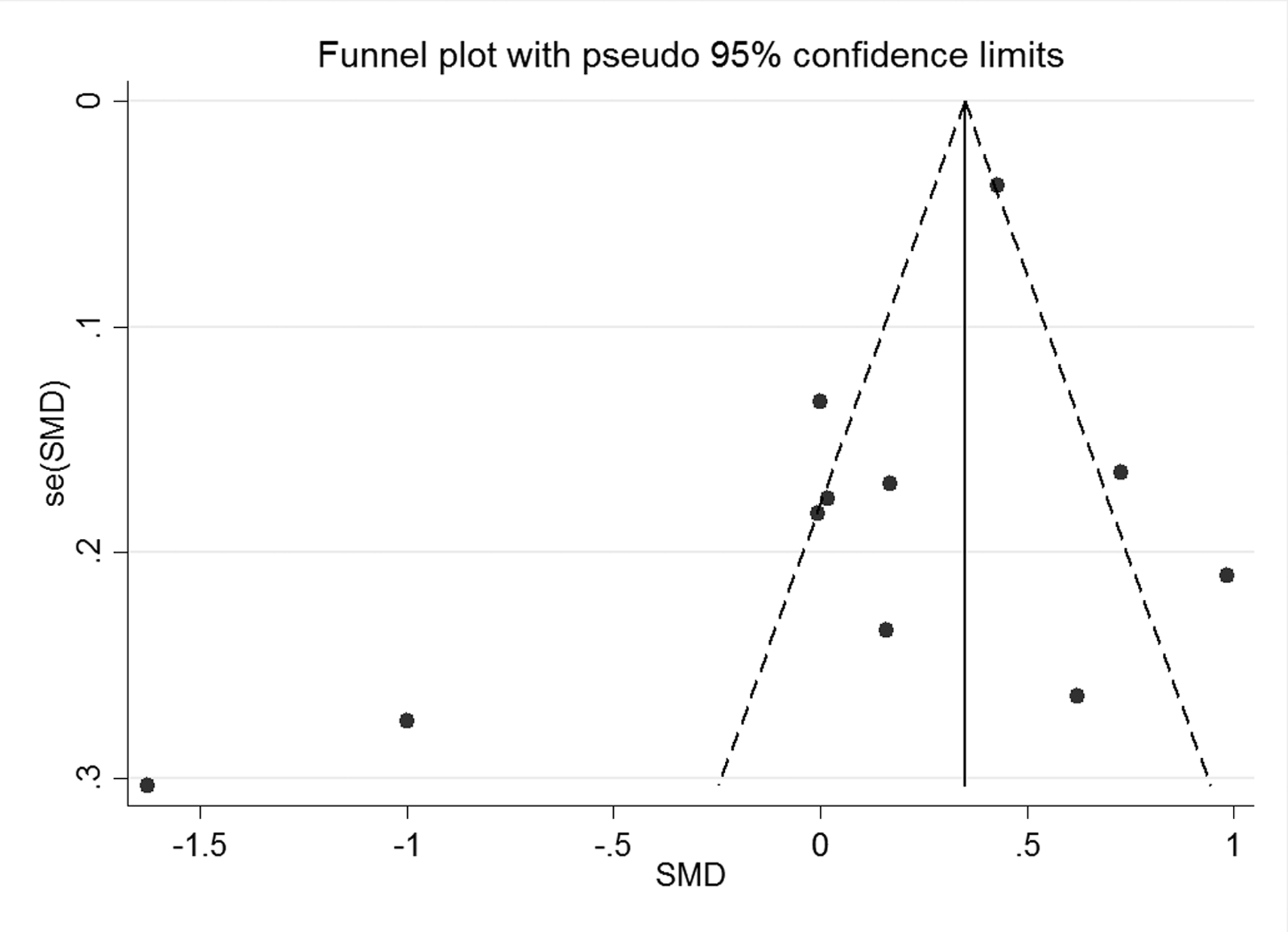

Supplement: Supplementary file 5 — Figure S3. Begg’s funnel plot of included studies for potential publication bias between nonalcoholic fatty liver disease (NAFLD) patients and controls. SMD, standardized mean differences. (TIFF 111 kb) [file 12944_2017_566_MOESM5_ESM.tif]
